# The Week's Work

**Published:** 1911-02-25

**Authors:** 


### The Week's Work.

### CASUALTY DEPARTMENTS.

The casualty departments of our hospitals have often been a source of some anxiety to the committees and governing bodies of those institutions. The work done in casualty departments looms large in the public eye, and criticism of incidents occurring therein is often hasty and disproportionate. In large hospitals, of course, this department is run by special officers, who are able to devote their whole attention to the work; but in many places the amount of casualty work is insufficient to warrant the appointment of a special casualty officer, and the department is often looked after by one of the resident medical officers. A little consideration shows that such a system is unfair, both to the resident and to the public, in any but small hospitals in quiet towns. Apart from the fact that sooner or later some delay in attending to a case will be the cause of complaint, it is manifestly difficult for a resident to call to his aid that care, tact, and discrimination which are so essential in casualty work, when he has just been interrupted while engaged on other work. The system is unavoidable in certain circumstances, but wherever possible has been obviated by more efficient methods.

### THE LONDON SYSTEM.

In the Metropolis there are scarcely any general hospitals where there is not a special casualty officer, resident or otherwise. Some of these appointments have not been made without a certain amount of opposition on the part of those residents who had previously shared in this work; for the latter fully appreciate the importance and the ultimate usefulness to themselves of this experience, and are naturally adverse to being relieved of it entirely. An excellent compromise has been found recently at one hospital, with the object of eliminating the above-mentioned disadvantage. By the simple method of appointing their residents for seven months instead of for six, the authorities are enabled to have the unfettered services of a casualty officer, and each of the three house physicians and three house surgeons have the opportunity of acquiring the excellent experience which this work entails for one month in turn, without the distractions of ward work; moreover, each man has the opportunity of doing both medical and surgical work during his last month as resident. In this manner not only is the casualty department looked after by a more or less experienced officer at a nominal cost to the hospital, but the resident's appointment is also considerably enhanced in value. From the point of view of hospital finance it undoubtedly pays to treat with the utmost consideration those patients coming under the somewhat elastic definition of "casuals," provided always that the questions of "hospital abuse" and the interests of local practitioners are duly attended to. It is also necessary to guard against the practice of some of the more knowing casual "chronics," who will present themselves at times convenient to themselves on purpose to avoid a long wait in the out-patients' department, in which their not urgent complaints should properly be investigated.

### PROGRESS IN THE WEST RIDING.

The West Riding of Yorkshire is, on the whole, taking up questions of hospital reconstructions and increased support with much vigour, and Batley has just stepped into line with the many other cities and towns in the country whose memorial to the memory of the late King is taking the shape of assistance to local hospitals. At a town's meeting held last week the Mayor (Alderman John Stubley) voiced a feeling which existed in certain quarters that a fitting way of commemorating the reign of King Edward would be to increase the permanent endowment fund of the Batley Hospital and liquidate a debt of some hundreds of pounds on the extension fund. Our readers will probably be aware that the hospital was enlarged a few years ago, but despite this enlargement its work is constantly expanding. The endowment fund of the institution now stands at about £6,700, and this, in the opinion of those who are intimately connected with the work of the hospital and also with its needs, is altogether inadequate. The sum mentioned, it is felt, ought to be increased to £10,000 or £12,000 at the very least. How near to this desired total sum the local fund which the meeting decided should be opened will come remains to be seen, but the people of Batley will doubtless contribute generously to a memorial, the usefulness of which will most certainly appeal with force to the workers in the Heavy Woollen District.

### THE WEEK'S DRUG MARKET.

The drug market continues active, and although actual price changes have been fewer in number, the advancing tendency which has been so evident during the past few weeks continues. Cod liver oil is much dearer in consequence of unfavourable reports from Norway as to the fishing, the number of cod caught up to date and the yield of oil being very substantially less than at the same time last year. Should the weather in the fishing grounds improve, we may hear better reports, but until then prices are likely to advance still higher. Camphor and menthol have not been in such demand as they were recently, but values are maintained. Opium is still inclined dearer, and the prices of morphine and apomorphine have been advanced; a rise in the quotation for codeine is considered very probable. Quicksilver has again been advanced 10*s*. per bottle and a further advance is not unexpected. Makers of calomel, corrosive sublimate and other salts of mercury have added another penny per lb. to their prices. Cassia oil and oil of cloves are dearer. Saffron is slightly higher in price. Carbolic acid has ceased to advance in value and makers are increasing their production.

